# Editorial: Curriculum Applications in Microbiology: Bioinformatics in the Classroom

**DOI:** 10.3389/fmicb.2021.705233

**Published:** 2021-07-01

**Authors:** Melanie Crystal Melendrez, Sophie Shaw, C. Titus Brown, Brad W. Goodner, Christopher Kvaal

**Affiliations:** ^1^Anoka-Ramsey Community College, Cambridge, MN, United States; ^2^Centre for Genome Enabled Biology and Medicine, University of Aberdeen, Aberdeen, United Kingdom; ^3^Department of Population Health and Reproduction, University of California, Davis, Davis, CA, United States; ^4^Hiram College, Hiram, OH, United States; ^5^Department of Biology, St. Cloud State University, St. Cloud, MN, United States

**Keywords:** bioinformatics and computational biology, undergraduate research, microbiology, curriculum—undergrad and postgrad, software, genomics, science technology engineering mathematics

John Naisbitt stated in his 1982 book Megatrends, “We are drowning in information but starved for knowledge.” The statement, made nearly 40 years ago, seems acutely applicable in today's scientific and academic world. Reviews by Barba et al. and van Dijk et al., provide a nice historical perspective on the growth of sequencing technology. Over three decades, sequencing technology has improved greatly from 1987 when the first ABI automated sequencing machine went to market up through the mid 2010s when next generation sequencing platforms from 454 Life Sciences, Illumina and other companies were outputting up to 1,800 Gb per run (Barba et al., 2014; van Dijk et al., 2014). Technology has since progressed even further with the development of long-read and single molecule (Pacific Biosciences, Illumina, Oxford Nanopore, and 10X Genomics) sequencing systems that can output terabytes of data, per run, in a matter of days (van Dijk et al., 2018). Specifically, in the areas of genomics, proteomics, and transcriptomics, we are now producing upwards of 1 zetta-bases/year (Stephens et al., 2015). The explosion of data has increased the demand for hardware and software development to manage and analyze the data as well as qualified personnel in bioinformatics to sift through the outputs to draw meaningful conclusions. A report from Reports and Data states the global bioinformatics market is projected to reach 18.96 billion USD by 2026 (Reports and Data, 2019) and this means re-thinking not only how we store data but how we train the next generation of scientists. The greatest needs identified in various surveys compiled by the NSF, ELIXIR-UK, and EMBL-ABR include: (i) data quality and control (ii) data analysis skills in visualization and interpretation, (iii) data mining, manipulation and management, (iv) analysis reproducibility, and (v) statistics (Kanwal et al., 2017; Kim et al., 2018; Attwood et al., 2019).

The large quantity of data available for analysis in many scientific fields is both a strength and a weakness in bioinformatic analysis. There are several databases and repositories available to acquire sequence data (INSDC: ENA+DNA Data bank of Japan and Genbank SRA, GISAID) and it is essential to know that not all data may be handled the same way. This variability in the quality of data that is released to scientists and the public at large can result in low quality data being analyzed and potentially spurious conclusions. A prime example can be seen in the current pandemic where SARS-CoV-2 sequences can be obtained from several different databases and analyzed in real-time. In the haste to make genomes available, the quality of what is released has been variable with challenges in consistent nomenclature (Gozashti and Corbett-Detig, 2021), and genomes containing errors created by sequencing artifacts, sample preparation, consensus calling approaches, or contamination (De Maio et al., 2020a,b; Van Noorden, 2021). Analysis of these genomes, even among experts, can lead to data misinterpretation, over-interpretation, and confusion on important topics such as SARS-CoV-2 origins (Andersen et al., 2020; Zhang et al., 2020; Wacharapluesadee et al., 2021). However, it is important to recognize these challenges to big data quality control, management, analysis, and reproducibility are not unique to SARS-CoV-2 but are systemic in many subfields of bioinformatics such as microbiome analysis (Katsnelson, 2019), metatranscriptomics (Shakya et al., 2019), and RNA-seq (Simoneau et al., 2021) analysis.

Recognition of these short-comings of big data acquisition, quality control, reproducibility, management, and analysis across bioinformatics disciplines have led to improvements in next generation sequencing workflows and quality control (Charre et al., 2020; De Maio et al., 2020b; Van Damme et al., 2021), efforts to use provenance, github, and docker containers to facilitate reproducibility (Kanwal et al., 2017; Kulkarni et al., 2018; Menegidio et al., 2018; Bolyen et al., 2019; Wercelens et al., 2019), nomenclature clarification (Rambaut et al., 2020, 2021), an increased emphasis on workflow automation (Reiter et al., 2021), and database curation and consolidation (Heard et al., 2021). As the tools and refinements to how scientists manage and analyze data continue to move forward, the demand for qualified big data analysts, statisticians, and bioinformaticians is increasing rapidly (Gómez-López et al., 2019; Terry, 2019; Tammi et al., 2020).

To address the need for big data management and analytical skill sets, many university programs have emerged offering certificates, Master's degrees and even Ph.D. degrees in the field of bioinformatics. The most recent guidance on bioinformatics core competencies has highlighted the importance of developing informatics skill sets early in the undergraduate curriculum (Welch et al., 2014; Vincent and Charette, 2015; Mulder et al., 2018; Wilson Sayres et al., 2018; Tractenberg et al., 2019). However, few curricula at the undergraduate level introduce big data analytics and bioinformatics systematically and many students graduate without a full understanding of what bioinformatics is or how it can be used to solve biological problems.

Several bioinformatic disciplines: i.e., metagenomics, genome construction/annotation, pathogen discovery, phylogenetics, metabolomics, and transcriptomics, have well-known workflows that teach valuable skills in data management, analytics, interpretation, and troubleshooting, but have yet to be translated to the classroom. Additionally, while many microbiology instructors recognize the importance of integrating more research, real-world datasets, and informatics into the classroom, they feel their training is inadequate, their curriculum is already over-full, or students do not appear particularly interested or prepared for such topics in the course (Williams et al., 2019). For many instructors, it can be daunting to put together bioinformatics curriculum modules if you are not familiar with the software or general topics within bioinformatics that students can explore.

This Research Topic focuses on bringing both research and educational communities together; encouraging researchers to translate their studies and pipelines into teaching tools and curriculum, and encouraging educators to dive into messy real-world datasets when teaching microbiology. Much of the challenge in implementing research or bioinformatics focused modules in the undergraduate classroom revolves around implementation. Bennet discusses strategies for blending your classroom to incorporate undergraduate research and bioinformatics modules into your curriculum design (CURE). Bennet takes a “workshop” or “project-based” approach to introduce the often complicated and challenging topic of RNA-Seq analysis (Conesa et al., 2016; Bennett) and discusses the long term outcomes for students experiencing this particular CURE as well as educational applications.

Another challenge in implementation of bioinformatics workflows in the classroom is the requirement for background experience in a variety of topics, both biological and computational. While many biology instructors are comfortable introducing and expanding on biological topics related to research and design, they are less comfortable discussing the computing aspects of bioinformatic analysis such as coding languages, data quality control, and data management. Several papers in the special topic discuss data workflows that utilize Microsoft Excel (Mitchell et al.; Hankey et al.; Kruchten). While many individuals working in advanced bioinformatic analysis may cringe at the idea of excel data analysis and tables, this program is well-used in classrooms globally and many instructors are comfortable with implementing data analysis and mathematical functions in the Excel environment. Programs such as Excel can provide a bridge between the user-friendly, GUI-based interfaces and the world of command-line applications (CLI). Krutchen, in particular, offers a nice comparison of the use of Excel vs. the R statistical language when analyzing metagenomic datasets and this may serve as motivation for instructors to explore other programming and CLI-based workflows (Kruchten). Topic papers in the methods category show instructors how to introduce, discuss, and/or implement coding languages and CLI-based bioinformatics in their classroom such as python/R for microbiome analysis (Rosen and Hammrich), basic command line proficiency in analyzing genome scale data for microbial isolates (Petrie and Xie), and how to conduct metagenomic analysis using the R statistical package (Kruchten) or QIIME, which contains its own language and syntax for implementation (Bolyen et al., 2019; Rosen and Hammrich).

Additional topic papers contain curricular designs for introducing and teaching a variety of bioinformatic analysis skills in the classroom without the need for teaching additional modules on coding skills. Topic papers discuss gene discovery and genome annotation using a variety of free web-accessible programs (Amatore et al.; Koury et al.; Martins et al.), microbiome analysis using PUMAA (Mitchell et al.), 16S amplicon identification using DNALC and NCBI-BLAST databases and the DNA Subway software program (Tawde and Williams; Williams et al., 2014), metagenomics analysis using MG-RAST and the MicrobiomeAnalyst program (Meyer et al., 2019; Chong et al., 2020; Baker et al.), phage hunting using PHASTER and iTOL programs (Arndt et al., 2019; Letunic and Bork, 2019; Martinez-Vaz and Mickelson), and Cancer data analysis using The Cancer Genome Atlas (TCGA; Hankey et al.).

To account for variable quality of datasets analyzed in the classroom, special topic studies used already published, curated, data from the Cancer Genome Atlas or GENI-ACT toolkit (Hankey et al.; Koury et al.) or pre-curated genomes for genome prediction exercises rather than raw data from databases (Martins et al.). Studies that made use of raw data or minimally curated data utilized embedded quality assessment tools and discussion modules on data cleanup within their curriculum methods and workflow (Amatore et al.; Kruchten; Tawde and Williams; Petrie and Xie; Baker et al.; Mitchell et al.). However, discussion and training on quality and data management needs to be ongoing; especially given data is being reused for educational purposes. Wilkinson and colleagues proposed the FAIR guiding principles to support the accessibility, findability, interoperability, and reusability of data in science (FAIR principles for data stewardship, 2016; Wilkinson et al., 2016) and there are workshops available on how to get started with “FAIR data” (https://mdibl.org/course/applied-bioinformatics-2021/). These principles should be considered widely in addition to the use of provenance and contained workflows or containers such as those mentioned earlier. In the overwhelming world of big data analysis it will be important for instructors to translate complex analysis techniques to their novice students; a key challenge is balancing quality and rigor with simplicity.

Finally the topic papers extend into existing scientific communities, where skills needed for data analysis are lacking by a large number of current researchers and professionals tasked to conduct bioinformatics analysis and interpretation. Therefore, workshops to educate existing researchers and laboratory personnel, from the level of graduate student to principal investigator, have become more frequent. These professional development and “train the trainer” workshops are attractive in that they are intensive short term experiences that teach very specific skill sets related to computational jobs in the field (McGrath et al., 2019). The Physalia courses (https://www.physalia-courses.org/), Cold Spring Harbor Laboratory Short Courses (https://meetings.cshl.edu/courses.html), and various workshops offered by the Evolution and Genomics training team (http://evomics.org/workshops/) and the MDI Biological Laboratory (https://mdibl.org/course/bioinformatics-t3-2021/) are a few examples of training experiences that undergraduates, graduates and professional personnel can use to augment their skill sets in the field of genetic analysis and computational biology. Internationally, these short term intensive educational opportunities, putting bioinformatics in the classroom, have proven useful in bringing staff and personnel up to date on the latest technologies and analysis capabilities to increase job performance and institute mission output. The BioCANET network in Central America (Orozco et al., 2013), Walter Reed Army Institute of Research (WRAIR) in South America (Pollett et al., 2016), H3Africa consortium in Africa (Aron et al., 2017; Ahmed et al., 2018; Shaffer et al., 2019), and APBioNet in Asia (Khan et al., 2013; Ahmad et al., 2019) are all aimed at increasing capacity for educational and research institutions in the areas of data management, systems administration, biostatistics, genome wide association studies, next generation sequencing analysis, metagenomics, and virology; and all have had success using this educational format. Our topic supports this educational “workshop” format of continued training for professional personnel through a paper by Maljkovic Berry et al., on implementation of a bioinformatics workshop for laboratory and research personnel at a US Department of Defense laboratory located in Kisumu, Kenya.

Special topic papers detail curriculum set up and implementation of bioinformatics modules or coding contain supplemental material to facilitate readers in their own implementation of the module or curriculum design in their classroom. We hope to convey through this topic the versatility of instructional designs that can be used to teach students at all levels of expertise, from high school to established professionals, how to leverage the strength of coding, software, and computational analysis to accomplish their research goals and further scientific teaching and discovery.

## Author Contributions

MM contributed to the initial concept of the special topic and wrote the initial draft of the editorial. All authors contributed to the design and proposal of the special topic, participated as active guest editors to manage manuscripts and oversee the special topic, and contributed to editorial revision and final approval for submission.

## Conflict of Interest

The authors declare that the research was conducted in the absence of any commercial or financial relationships that could be construed as a potential conflict of interest.
